# Presymptomatic Awareness of *BRCA1/BRCA2* Status and Outcomes in Women With Ovarian Cancer

**DOI:** 10.1001/jamanetworkopen.2025.1435

**Published:** 2025-03-21

**Authors:** Shunit Armon, Adi Hollander, Yakir Segev, Ora Rosengarten, Ariela Tomer, Ephrat Levy-Lahad, Rachel Michaelson-Cohen

**Affiliations:** 1 Department of Obstetrics and Gynecology, Shaare Zedek Medical Center, Jerusalem, Israel; 2 The Eisenberg R&D Authority, Shaare Zedek Medical Center, Jerusalem, Israel; 3 Faculty of Medicine, The Hebrew University of Jerusalem, Jerusalem, Israel; 4 Department of Internal Medicine, Hadassa Medical Center, Jerusalem, Israel; 5 Department of Gynecologic Oncology, Carmel Medical Center, Bruce Rappaport Faculty of Medicine, Technion Institute of Technology, Haifa, Israel; 6 The Helmsley Cancer Center Medical Center, Shaare Zedek Medical Center, Jerusalem, Israel; 7 The Fuld Family Medical Genetics Institute, Shaare Zedek Medical Center, Jerusalem, Israel

## Abstract

This cohort study examines the association between patients’ prediagnosis awareness of their genetic risk for ovarian cancer and cancer outcomes.

## Introduction

Carriers of germline *BRCA1/BRCA2* pathogenic variants (*BRCA* PVs) have increased risk of ovarian cancer (OC). Risk-reducing bilateral-salpingo-oophorectomy (RR-BSO), associated with lower OC risk and all-cause mortality, is recommended at age 35 to 40 years for *BRCA1* and 40 to 45 years for *BRCA2* carriers.^1,2^ However, *BRCA*-PV carriers are often identified only after cancer diagnosis since approximately half lack relevant family cancer history.^3^ Mean uptake of RR-BSO is 64% and only 31.3% of patients undergo RR-BSO at the recommended age, mostly due to delayed genetic diagnosis.^4^ Awareness of *BRCA*-PV carrier status before breast cancer diagnosis is associated with better overall survival (OS).^5^ We aimed to evaluate the association between prior *BRCA*-PV carrier status knowledge and OC outcomes.

## Methods

This retrospective cohort study extracted medical records data (2000-2023) from Shaare Zedek Medical Center and Carmel Medical Center. Patients (all women) received guideline-based surveillance and prevention recommendations, including RR-BSO. The Shaare Zedek and Carmel Medical Centers’ Institutional Review Boards approved the study and waived informed consent because deidentified data were used. We followed the STROBE reporting guideline.

Unpaired, 2-tailed *t* test and χ^2^ test were used for continuous and categorical variables, respectively; logistic regression was used for multivariate analyses. Kaplan-Meier analysis was performed, log-rank test examined differences between survival curves, and Cox regression calculated hazard ratios (HRs). Two-sided *P* < .05 indicated statistical significance. Data were analyzed between April and August 2024 using SPSS 25 (IBM) and R 4.3.2 (RStudio).

## Results

We identified 132 germline *BRCA1/BRCA2*-PV carriers diagnosed with OC (mean [SD] age at diagnosis, 56.9 [11.2] years) (Table). Of these patients, 34 were aware of their carrier status before OC diagnosis (prediagnosis awareness) and 98 had postdiagnosis awareness. Prediagnosis awareness was associated with earlier stage at diagnosis compared with postdiagnosis awareness (stage I: 10 [32.3%] vs 6 [6.3%]; *P* = .001). Multivariate analysis (controlling for age, year of diagnosis, family history, and gene) indicated postdiagnosis awareness was associated with more advanced stage at diagnosis (odds ratio, 4.35; 95% CI, 1.49-12.72; *P* = .007). Downstaging in prediagnosis awareness was reflected in lower CA-125 levels (mean [SD], 346 [441] vs 1749 [2920] IU; *P* < .001), fewer ultrasonographic findings at diagnosis (16 of 21 [76.2%] vs 75 of 79 [94.9%]; *P* = .02), more diagnoses through surveillance (5 of 25 [20.0%] vs 6 of 44 [13.6%]; *P* < .001) and RR-BSO (11 of 25 [44.0] vs not applicable; *P* < .001), and lower rates of second-line treatment (8 of 15 [53.4%] vs 36 of 49 [73.5%]; *P* = .01). Both groups had similar rates of optimal debulking, recurrence, poly (ADP-ribose) polymerase inhibitor treatment, and OS. In univariate analysis, groups did not differ in disease-free survival (DFS) (Figure); in multivariate analysis, HR for DFS between groups was 0.38 (95% CI, 0.18-0.83; *P* = .02).

**Table.** Comparison of Patients With Prediagnosis vs Postdiagnosis Awareness of *BRCA* Pathogenic Variant Carrier Status

Characteristic	Patients, No./total No. (%)			*P* value
	Prediagnosis awareness (n = 34)	Postdiagnosis awareness (n = 98)^a^	All (N = 132)	
Age at OC diagnosis, mean (SD), y	54.7 (10.1)	57.7 (11.5)	56.9 (11.2)	NS
Period of diagnosis				
2000-2010	14/34 (41.2)	43/98 (43.9)	57 (43.0)	NS
≥2011	20/34 (58.8)	55/98 (56.1)	75 (56.8)	
Family history score^b^				
None-low	4/27 (14.8)	20/62 (32.3)	24 (26.9)	NS
Moderate-high	23/27 (85.2)	42/62 (67.7)	65 (73.0)	
Gene				
* BRCA1*	20/34 (58.8)	71/98 (72.4)	91 (68.9)	NS
* BRCA2*	14/34 (41.2)	27/98 (27.6)	41 (31.1)	
Genetic variant^c^				
*BRCA1* 185delAG	16/30 (53.3)	44/85 (51.8)	60 (52.2)	.04
*BRCA1* 5382insC	7/30 (23.3)	8/85 (9.4)	15 (13.0)	
*BRCA2* 6174delT	7/30 (23.3)	20/85 (23.5)	27 (23.5)	
Other^d^	NA	13/85 (15.3)	13 (11.3)	
Method of diagnosis				
Symptoms	8/25 (32.0)	35/44 (79.5)	43 (62.3)	<.001
Incidental^e^	1/25 (4.0)	3/44 (6.8)	4 (5.8)	
RR-BSO	11/25 (44.0)	NA	11 (15.9)	
Surveillance	5/25 (20.0)	6/44 (13.6)	11 (15.9)	
Histological type				
Serous	22/31 (71.0)	73/89 (82.0)	95 (79.2)	.03
Endometrioid	3/31 (9.7)	NA	3 (2.5)	
Other^f^	6/31 (19.4)	16/89 (18.0)	22 (18.3)	
Stage at diagnosis^g^				
I	10/31 (32.3)	6/95 (6.3)	16 (12.7)	.001
II	6/31 (19.4)	13/95 (13.7)	19 (15.1)	
III	11/31 (35.5)	62/95 (65.3)	73 (57.9)	
IV	4/31 (12.9)	14/95 (14.7)	18 (14.3)	
CA-125 level at diagnosis, IU				
Median (IQR)	219 (19-500)	590 (208-2042)	500 (130-1523)	<.001
Mean (SD)	346 (441)	1749 (2920)	1460 (2669)	<.001
>35	16/24 (66.7)	82/85 (96.5)	98 (89.9)	<.001
≤35	8/24 (33.3)	3/85 (3.5)	11 (10.1)	
Abnormal sonogram findings at diagnosis	16/21 (76.2)	75/79 (94.9)	91 (91.0)	.02
Primary treatment				
Primary surgery	25/30 (83.3)	65/94 (69.1)	90 (72.6)	NS
Neoadjuvant chemotherapy	5/30 (16.7)	27/94 (28.7)	32 (25.8)	
Palliative	NA	2 (2.1)	2 (1.6)	
Surgical results				
Optimal debulking	21/24 (87.5)	57/73 (78.1)	78 (80.4)	NS
Suboptimal debulking	3/24 (12.5)	16/73 (21.9)	19 (19.6)	
Chemotherapy				
Platinum + paclitaxel	23/28 (82.1)	82/90 (91.1)	105 (89.0)	NS
Single agent	4/28 (14.3)	8/90 (8.9)	12 (10.2)	
None	1/28 (3.6)	NA	1 (0.8)	
Second treatment line	8/15 (53.4)	36/49 (73.5)	44 (68.8)	.01
PARP inhibitors^h^	5/23 (21.7)	25/69 (36.2)	30 (32.6)	NS
Recurrence	14/27 (51.9)	57/88 (64.8)	71 (61.7)	NS
5-y Survival	20/25 (80.0)	51/68 (75.0)	71 (76.3)	NS

Abbreviations: CA-125, cancer antigen 125; NA, not applicable; NS, not significant; OC, ovarian cancer; PARP, poly (ADP-ribose) polymerase; RR-BSO, risk-reducing bilateral-salpingo-oophorectomy.

^a^ Mean (SD) time from diagnosis to testing was 8 (18) months.

^b^ Family history was categorized as none-low or medium-high by the Hereditary Breast and Ovarian Cancer Likelihood Score, as previously described.^2^

^c^ Distributions of *BRCA1* vs *BRCA2* pathogenic variant (PV) and specific PV were similar to the distributions described in a large Israeli cohort of *BRCA* carriers with OC.^5^

^d^ Other genetic variants included *BRCA1* 4572del22, *BRCA1* W1815X (5563 G>A), *BRCA1* p.P1812A, *BRCA1* c.5434C>G p.P1812A, *BRCA1* 2846del4, *BRCA1* del exon 14, *BRCA1* c.3901_3902delAG p.Ser1301Terfs*1 rs80357646, *BRCA2* c.6024dupG (6252inG), *BRCA1* 4153delA, *BRCA2* c.2957dupA: p.Asn986Lysfs*, *BRCA2* DEL EX1-18, *BRCA1* c.5434C>G;p.P1812A, *BRCA1* c.2934T>G (Y978X), and *BRCA2* c.3751dupA; p.Thr1251AsnfxX.

^e^ Incidental method: routine pelvic ultrasound, which was not part of surveillance recommended for *BRCA* PV carriers.

^f^ Other histologic types included mixed and unspecified.

^g^ Stage distribution by ascertainment for prediagnosis group: stage I included 5 of 9 and 3 of 9 cases diagnosed through RR-BSO and surveillance, respectively, and stage II to IV included 6 of 15 and 3 of 15 cases diagnosed through RR-BSO and surveillance, respectively. For the postdiagnosis group, stage I included 1 of 3 and 1 of 3 cases diagnosed through symptoms and surveillance, respectively, and stage II to IV included 34 of 41 and 5 of 41 cases diagnosed through symptoms and surveillance, respectively.

^h^ PARP inhibitors were introduced into clinical practice for recurrent disease in 2014.

**Figure.**
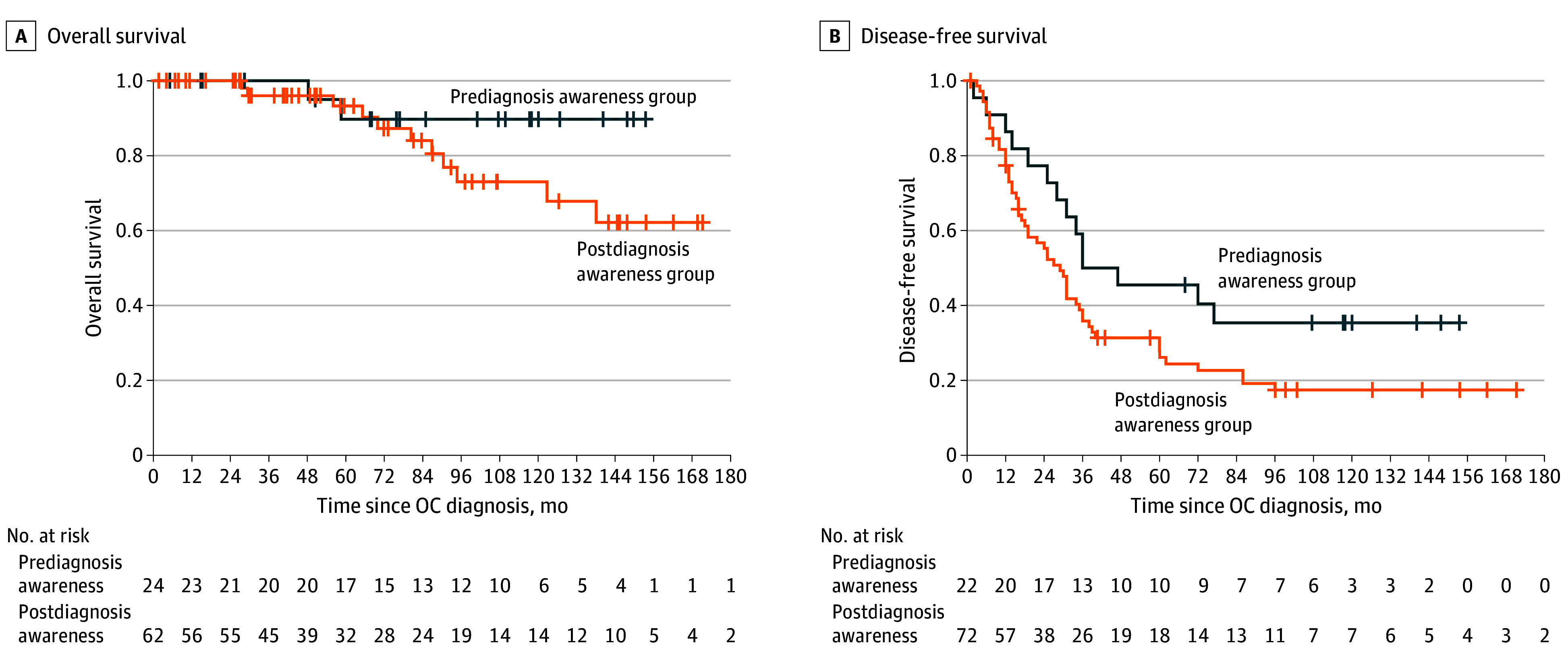
Kaplan-Meier Curves of Overall Survival (OS) and Disease-Free Survival (DFS) in *BRCA1* and *BRCA2* Carriers With *BRCA* Status Awareness Before or After Ovarian Cancer (OC) Diagnosis The onset of analysis was time of OC diagnosis. Vertical hatches represent censored individuals. Groups did not differ in OS (log rank, 0.5) or DFS (log rank, 0.07). However, when controlling for age, year of diagnosis, family history, and gene, the hazard ratio (HR) for DFS in the prediagnosis vs postdiagnosis awareness groups was 0.38 (95% CI, 0.18-0.83; *P* = .02).

RR-BSO was recommended to all carriers. However, among 34 patients with prediagnosis awareness, 27 (79.4%) had genetic testing only after age 40 years; 13 were diagnosed before or during RR-BSO or by surveillance; and 2 had primary peritoneal carcinoma after RR-BSO. Six patients presented with symptoms; 6 lacked information regarding diagnostic method.

## Discussion

The findings on *BRCA*-PV carriers diagnosed with OC reflect older age at genetic testing and RR-BSO deferral. Prediagnosis awareness of *BRCA* status was associated with earlier stage at diagnosis, fewer second-line treatments, and longer DFS. No significant OS advantage was observed, which could be explained by limited sample size but may be consistent with observations that, in the general population, OC downstaging does not translate to decreased mortality.^6^

Study limitations include retrospective design, selection of patients with OC who were genetically tested, and sample size. Prediagnosis and postdiagnosis awareness groups were similar in age, family history, and year of diagnosis, suggesting these groups are comparable.

Most patients with prediagnosis awareness had genetic testing only after age 40 years; all occult cancer cases were detected from RR-BSO performed after age 54 years. This large proportion of potentially preventable cancers further supports *BRCA1/BRCA2* screening before age 40 years in populations with high carrier rates.

## Notes

Supplement.
Data Sharing Statement
