# Supplementary material for: Presymptomatic Awareness of BRCA1/BRCA2 Status and Outcomes in Women With Ovarian Cancer
Source: JAMA Netw Open. 2025 Mar 21;8(3):e251435. doi: 10.1001/jamanetworkopen.2025.1435 (PMC11929032; doi:10.1001/jamanetworkopen.2025.1435)
Supplement: Supplement. — Data Sharing Statement [file jamanetwopen-e251435-s001.pdf]

Data Sharing Statement

Armon. Presymptomatic Awareness of BRCA1/BRCA2 Status and Outcomes in Women With Ovarian Cancer. *JAMA Netw Open*. Published March 21, 2025.
doi:10.1001/jamanetworkopen.2025.1435

Data
Data available: Yes
Data types: Deidentified participant data
How to access data: shunita@szmc.org.il
When available: With publication

Supporting Documents
Document types: Other (please specify)
Additional Information: Deidentified data and statistical analysis
How to access documents: shunita@szmc.org.il
When available: With publication

Additional Information
Who can access the data: researchers whose proposed use of the data has been approved
Types of analyses: for a specified purpose
Mechanisms of data availability: after approval of a proposal
